# Evidence of interactions between white sharks and large squids in Guadalupe Island, Mexico

**DOI:** 10.1038/s41598-020-74294-4

**Published:** 2020-10-13

**Authors:** Edgar E. Becerril-García, Daniela Bernot-Simon, Marcial Arellano-Martínez, Felipe Galván-Magaña, Omar Santana-Morales, Edgar M. Hoyos-Padilla

**Affiliations:** 1 Instituto Politécnico Nacional, Centro Interdisciplinario de Ciencias Marinas, 23096 La Paz, Mexico; 2 Pelagios Kakunjá A.C., 23060 La Paz, Mexico; 3 Universidad Autónoma de Baja California Sur, 23080 La Paz, Mexico; 4 Universidad Autónoma de Baja California, 22860 Ensenada, Mexico; 5 Ecología Cielo Mar y Tierra A.C., 22880 Ensenada, Mexico; 6 Fins Attached Marine Research and Conservation, Colorado Springs, 80908 USA

**Keywords:** Animal behaviour, Ichthyology, Behavioural ecology, Marine biology

## Abstract

Shark-cephalopod interactions have been documented in trophic ecology studies around the world. However, there is little information about the encounters between white sharks *Carcharodon carcharias* and squids in the eastern North Pacific Ocean. Here we provide evidence of interactions between white sharks and large squids in the waters of Guadalupe Island, Mexico. Through the use of non-invasive techniques, we found the presence of evident scars made by large squids on the body of the white sharks, mainly on the head and trunk, of at least 14 sharks recorded during August–December in the years 2008, 2012, 2013, 2017 and 2019. The mean length of the white sharks was 3.7 m (SD ± 0.6; total length), although the majority of the sharks with scars were adult and subadult males (n = 9; 64%). One of these males was photographically recaptured during the same season in which the individual showed new scars, confirming that the squid-white shark interaction likely occurs near Guadalupe Island. Our results highlight the importance of the twilight zone for white sharks and the use of shared habitat and trophic interactions between squid and white sharks, in which future ecosystem studies should consider both species for management and conservation purposes.

## Introduction

The interactions between sharks and cephalopods are fundamental in coastal and oceanic ecosystems^1–3^. Cephalopods can represent up to 98% of the diet biomass of species such as the blue shark *Prionace glauca*, the scalloped hammerhead *Sphyrna lewini* (68%), or the pelagic thresher *Alopias pelagicus* (69%), because they provide nutrients in terms of proteins, carbohydrates and fatty acids^3,4^. In some species such as the white shark *Carcharodon carcharias*, previous studies have suggested that cephalopods are an important component of their diet, since large amounts of squids have been found in their stomachs^2,4^, and are reflected in the isotopic values of their muscle^5^.

In the eastern North Pacific Ocean, the white sharks evidence migratory patterns that involve their presence in an oceanic zone known as the Shared Offshore Foraging Area (SOFA) from February through June^6,7^, and in the waters surrounding Guadalupe Island from July to February^8,9^, with some sharks moving to Hawaii, the coast of Baja California and the Gulf of California^10,11^. Although feeding is not documented, the vertical movements of white sharks in deep waters have been identified as potential foraging behaviour by diving at average depths of 442–498 m in the SOFA^7^, and > 300 m in Guadalupe Island^9^. Both SOFA and Guadalupe Island have been suggested as areas with a high diversity of potential prey, in which cetaceans, sharks, bony fish, and cephalopods seasonally occur^7–9,12–14^.

Within the distribution of white sharks in the eastern North Pacific Ocean, there are important areas for the occurrence of large squid species such as the neon flying squid *Ommastrephes bartramii*, purpleback squid *Sthenoteuthis oualaniensis*, sharpear enope squid *Ancistrocheirus lesueurii*, jumbo squid *Dosidicus gigas*, and probably the giant squid *Architeuthis dux*^3,10,12,15,16^. However, scientific research has focused mainly on fishing activities near the coast where a greater catch of *D. gigas* has been reported during the months of July–December, which coincides with the white shark sighting season in Guadalupe Island^8,9,16^. Nevertheless, the studies involving white sharks and squids are scarce, so there is a lack of information regarding these interactions in the eastern North Pacific. The aim of the present study is to provide evidence of the interaction of large squids and white sharks in Guadalupe Island in order to highlight the importance of squid as potential prey in subadult and adult stages of white sharks.

## Results

**Figure 1:**
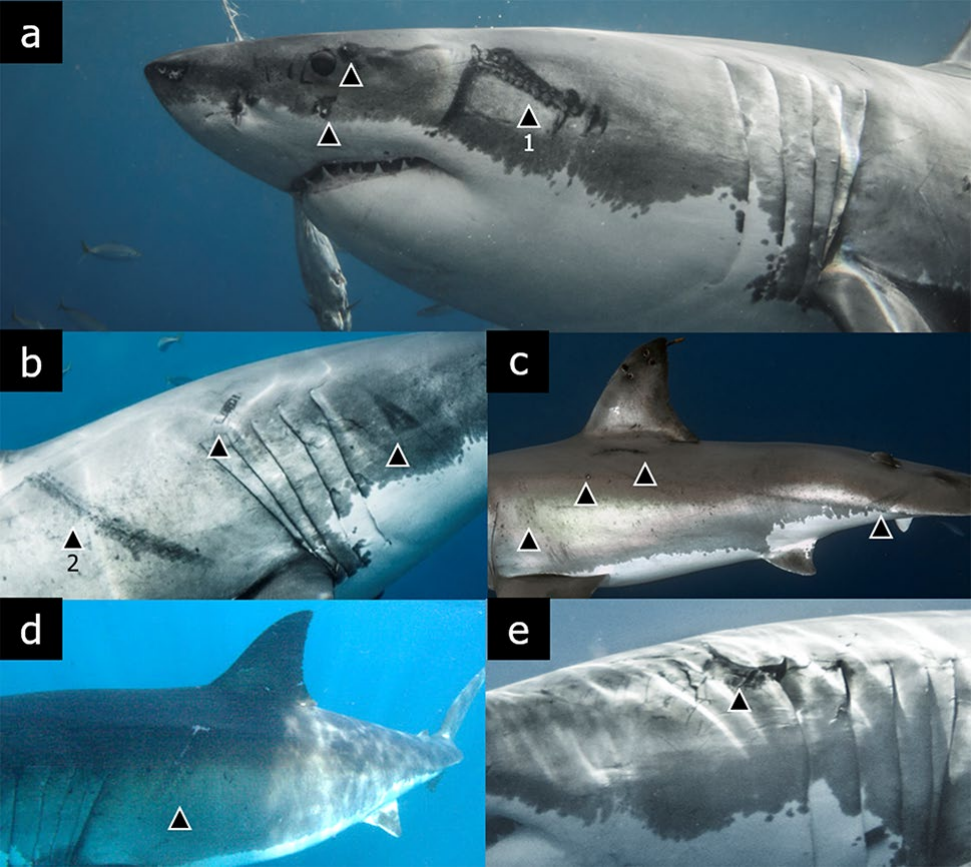
Some of the white sharks observed with squid scars (filled triangle) on their body in the waters surrounding Guadalupe Island, Mexico. WS11 (**a**,**b**); WS12 (**c**); WS01 (**d**); WS13 (**e**). Scars in which its length was estimated digitally (1,2).

A total of 14 white sharks were detected with unusual scars on their body during August–December of the years 2008, 2012, 2013, 2017 and 2019 (Table 1; Fig. 1). The average size (TL) of the white sharks was 3.7 m (SD ± 0.6), in which nine sharks (64%) were males (seven subadults; two adults) and five were females (36%; three subadults; two adults). Regarding sexual maturity, the majority of white sharks observed with scars were subadults (n = 10; 71%), although adults were also observed (n = 4; 29%). The shark WS11 was first observed with a scar on its trunk on 13 October 2017, which was detected again with the same scar during its recapture in August and September 2019. A few weeks later, this shark was registered with new scars that were first recorded in September, October and November (Fig. 1a,b), which indicates that this interaction likely occurred in Guadalupe Island between September 9–21 (Table 1).

**Table 1:** Records of white sharks with scars inflicted by squids in Guadalupe Island, Mexico.

Code	Sex	TL (m)	Maturity	Date	Area of the body
WS01	Female	4	Subadult	12/29/2008	Trunk
WS02	Male	3	Subadult	08/18/2012	Trunk
WS03	Male	3	Subadult	08/18/2012	Head and trunk
WS04	Male	3.5	Subadult	09/05/2012	Trunk
WS02	Male	3	Subadult	09/11/2012	Head and trunk
WS05	Male	3.5	Subadult	09/22/2012	Head and pectoral fin
WS06	Female	3.5	Subadult	10/24/2012	Trunk
WS07	Male	3	Subadult	08/25/2013	Trunk
WS08	Female	5	Adult	10/13/2013	Head
WS09	Male	4.5	Adult	10/30/2013	Head
WS10	Male	3	Subadult	08/10/2017	Head
WS11	Male	4	Adult	10/13/2017	Trunk^a^
WS12	Female	3.5	Subadult	11/02/2017	Head, trunk, and tail
WS11	Male	4	Adult	08/24/2019	Trunk
WS11	Male	4	Adult	09/09/2019	Trunk^a^
WS11	Male	4	Adult	21/09/2019	Trunk^a^ and head
WS11	Male	4	Adult	10/16/2019	Trunk^a^ and head
WS13	Female	4.5	Adult	11/09/2019	Head
WS11	Male	4	Adult	11/10/2019	Trunk^a^ and head
WS14	Male	3.5	Subadult	11/10/2019	Trunk

*TL* total length.

^a^Same scar observed after two seasons.

The scars observed on the white sharks were double or single layers with multiple sucker marks around the shark's head and in the trunk between the dorsal and pectoral fins (Fig. 1). Wounds near the shark's mouth and trunk suggest a defensive response from squid to the white shark, although this could not be confirmed. The pattern and shape of the scars matches the suction cups on the arms and tentacles of cephalopods as observed in other shark species^17^. The estimation of the diameter of the most evident sucker marks in the shark WS11 (Fig. 1a) allowed us to determine that the left side scar (≈ 20 cm long) had suction cups that measured 1–3 cm, while the length of the right side scar measured ≈ 60 cm long. Even though the cephalopod species could not be confirmed, observed marks from all analysed sharks could suggest an encounter with taxa such as the jumbo squid *D. gigas*, the neon flying squid *O. bartramii*, or the giant squid *A. dux* based on their potential distribution, arms and tentacles length, as well as the estimated size of the scars and suckers^12,16–18^.

## Discussion and conclusions

Guadalupe Island is an important feeding site for white sharks in the eastern North Pacific Ocean. The simultaneous presence of pinnipeds, yellowfin tuna (*Thunnus albacares*), other elasmobranchs and squids is an indicator of prey availability for the white shark during subadult and adult stages. Previous studies using stable isotopes have estimated the contribution of prey such as pinnipeds and *D. gigas* in the muscle of white sharks analysed in Guadalupe Island^5^; while this species of squid was also found as a prey item in the stomach of a 4.6 m female caught near California^4^. In the present study, the use of non-invasive techniques provided evidence of recent interactions between the white shark and large cephalopods. These observations highlight the importance of the mesopelagic zone for white sharks in terms of feeding, since subadult and adult sharks remain in deep waters below 100 m during the day^9,19^, similar to what has been recorded in other areas such as the SOFA^6,7^. In addition, the usual depth range of the probable species of cephalopods during daytime is 300–600 m for *O. bartramii*^20^ and 200–300 m for *D. gigas*^15^, which is consistent with the patrolling depths reported for the white shark in the area^7,9^ and coincides with the bathymetry of Guadalupe Island.

Squid interactions have been widely observed in other large marine predators such as sperm whales *Physeter macrocephalus*^21,22^. These interactions have been confirmed by the presence of squid beaks in the stomachs of sperm whales, in addition to the scars observed on the head and body of these cetaceans^21,22^. The fact that squid cause these marks on sharks suggests an extremely aggressive encounter between predator and prey, in which the defensive scars protrude on the head, gills and body of the white shark. The suction power of the arms and tentacles of large squids is likely to deform the structure of the shark dermal denticles and hence the scars, and in some cases generate open wounds depending on the intensity of the embrace^23^.

The consumption of cephalopods could be essential in the diet of the white shark by allowing a quick digestion and absorption due to the large amount of proteins and low lipid content present in this group of invertebrates^3,24–26^. In addition to the protein content, it has been suggested that some cephalopod species such as *D. gigas* contain high contents of essential fatty acids for shark reproductive processes, such as gonadal maturation and embryonic development, which could favour spermatogenesis in subadult males and pregnancy in adult females^24–27^.

The presence of scars caused by large squid in 14 sharks during the study period could seem a low number compared to the estimated number of white sharks in Guadalupe Island, which has been calculated to be 120 sharks^28^. However, the presence of scars could not be visible in all white sharks that prey on squid, as this will depend on the intensity of the interaction, effectiveness of the attack and the handling of the prey. The frequency of these interactions and their relevance in the trophic ecology of the white shark can be studied by tagging both sharks and squid, as well as complementary studies on fatty acids and stable isotopes of carbon and nitrogen^18,24,26^.

The white sharks from Guadalupe Island have a segregated migration in which subadult and adult males arrive in August and adult females in late September^8,9^. In this study, most squid scars were observed in subadult male sharks throughout the season, suggesting that their arrival to Guadalupe Island could be related to the consumption of squids and tunas, while the Northern elephant seals *Mirounga angustirostris* arriving in December would complement the diet of adult sharks in a dietary shift related to their maturity^4,5,9^. This emphasizes the importance of habitat use for both white sharks and squids, where future conservation-related studies should include the potential impacts of both prey and predators distribution, mainly in terms of oceanographical changes related to temperature, productivity and minimum oxygen layers^6,9,14–16,26^. This type of research will also be able to elucidate evolutionary aspects of the interaction between sharks and cephalopods, since both lineages are ancient and their study could provide relevant information on the ecological importance of both taxa in the dynamic exchange of matter and energy in marine ecosystems^6,12,18,26^.

## Materials and methods

**Figure 2:**
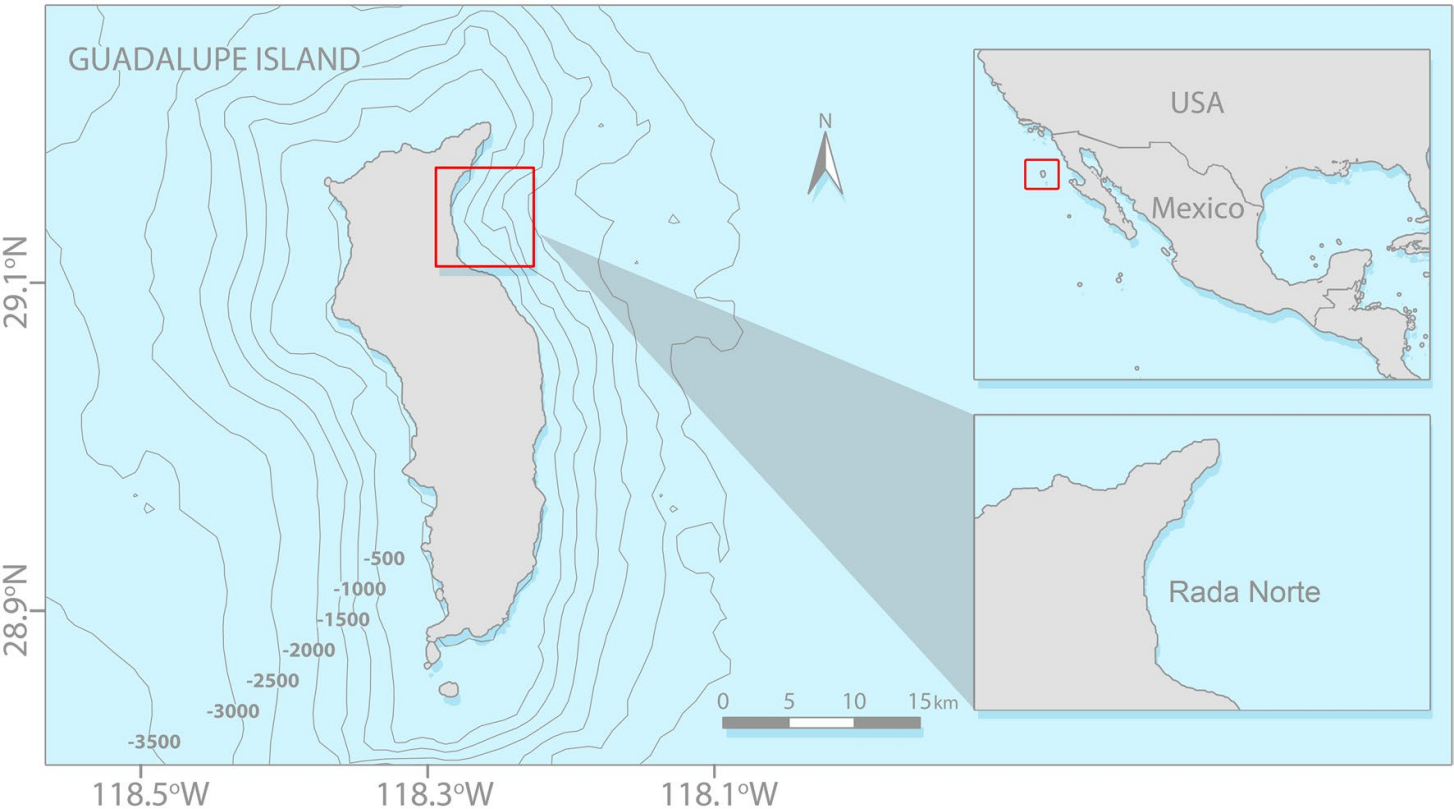
Location of Rada Norte Bay at Guadalupe Island, Mexico, with isobaths shown in meters (QGIS v3.10; Photoshop CC 2019 v20.0.7).

Guadalupe Island is located 240 km west from the closest point to the occidental coast of Baja California, Mexico (Fig. 2). This volcanic island is the nucleus of a biosphere reserve that presents a high diversity of seabirds, pinnipeds, bony fishes, elasmobranchs, marine invertebrates, and seaweeds^29^. As it is not on a continental shelf, the island has great depths near the coast, which exceed 1000 m of depth^29^. The average sea surface temperature is 18 °C with ranges between 16 and 20 °C, although maxima of up to 25 °C have been reported^29,30^. The island measures 32 km long and 6.5–9.5 km wide, although the white shark cage diving activity takes place in the northeast of the island in a bay known as Rada Norte (Fig. 2) mainly during the months of August–November^31^.

A set of approximately 2500 underwater pictures of white sharks were obtained during several expeditions onboard the tourist boats that visited the northern area of Guadalupe Island (Fig. 2) throughout August-December (2008–2019). The procedures for estimating total length, sex and photoidentification of white sharks were followed as indicated by Becerril-García et al.^31^, while the determination of sexual maturity was made according to Bruce and Bradford^32^. Obtained underwater photographs and videos were analysed, in which scars and unusual marks on the shark's body were observed. These photographs were classified according to the date and biological data of each white shark sighting. The photographic quality and appropriate angle of some pictures of the shark WS11, along with the data of its estimated size (total length), allowed the estimation of the length of the scars, as well as the diameter of the suckers in this individual using the AxioVision SE64 Rel. 4.9.1 software.

## Supplementary information

Supplementary Table 1.

## Data Availability

The dataset regarding the analysed white sharks is available as supplemental information (Table S1). Additional photographic records of the analysed white sharks are available from the corresponding author on request.
