# Supplementary material for: Evidence of interactions between white sharks and large squids in Guadalupe Island, Mexico
Source: Sci Rep. 2020 Oct 13;10:17158. doi: 10.1038/s41598-020-74294-4 (PMC7555532; doi:10.1038/s41598-020-74294-4)
Supplement: Supplementary file 1 — Supplementary Table 1. [file 41598_2020_74294_MOESM1_ESM.pdf]

Evidence of interactions between white sharks and large squids in Guadalupe Island, Mexico

Edgar E. Becerril-García[1,2], Daniela Bernot-Simon[3], Marcial Arellano-Martínez[1], Felipe Galván-Magaña[1], Omar Santana-Morales[4,5], Edgar M. Hoyos-Padilla[2,6*]

[1]Instituto Politécnico Nacional, Centro Interdisciplinario de Ciencias Marinas, La Paz, 23096, Mexico.
[2]Pelagios Kakunjá A.C., La Paz, 23060, Mexico
[3]Universidad Autónoma de Baja California Sur, La Paz, 23080, Mexico.
[4]Universidad Autónoma de Baja California, Ensenada, 22860, Mexico.
[5]Ecología Cielo Mar y Tierra A.C., Ensenada, 22880, Mexico.
[6]Fins Attached Marine Research and Conservation, Colorado Springs, 80908, USA.
*mauricio@pelagioskakunja.org

Table S1. Data set of the records of white sharks with squid scars on their body observed at Guadalupe Island, Mexico. Total Length (TL).

Code	Sex	TL (m)	Maturity	Date	Scar location and observations
WS01	Female	4	Subadult	12/29/2008	Left side: Long fresh scar in the trunk between dorsal and pectoral fin
WS02	Male	3	Subadult	08/18/2012	Scar on the right side of the body between the gills and dorsal fin
WS03	Male	3	Subadult	08/18/2012	Very long scar on the left side above gill slits
WS04	Male	3.5	Subadult	09/05/2012	Scar with sucker marks on the right side, between dorsal and pectoral fin
WS02	Male	3	Subadult	09/11/2012	Four scars on the left side of the body. Sucker marks between the eyes and gills
WS05	Male	3.5	Subadult	09/22/2012	Long scar in the right side behind the eye, and another one in the pectoral fin of the right side. Potential beak mark on the left side between eyes and gills.
WS06	Female	3.5	Subadult	10/24/2012	Scar in the trunk between dorsal and pelvic fin on the left side of the body
WS07	Male	3	Subadult	08/25/2013	Small scar on the right side of the body between the dorsal and pectoral fin
WS08	Female	5	Adult	10/13/2013	Top of the head, between eyes and gills
WS09	Male	4.5	Adult	10/30/2013	Top of the head, close to the gills
WS10	Male	3	Subadult	08/10/2017	Multiple scars mainly on the head, right side
WS11	Male	4	Adult	10/13/2017	Long scar on the right side (50 cm), between dorsal and pectoral fin
WS12	Female	3.5	Subadult	11/02/2017	Left side: Scars and beak marks below dorsal fin, another mark close to pectoral fin and near the anal fin.
WS11	Male	4	Adult	08/24/2019	Same long scar on the right side (50 cm; 2017), between dorsal and pectoral fin
WS11	Male	4	Adult	09/09/2019	Same long scar on the right side (50 cm; 2017), between dorsal and pectoral fin
WS11	Male	4	Adult	09/21/2019	Same long scar on the right side (50 cm; 2017), between dorsal and pectoral fin; new scar (triangle shape) on the right side, close to mouth, and another one up the gills. New and clear scar with sucker marks; left side
WS11	Male	4	Adult	10/16/2019	Same long scar on the right side (50 cm; 2017), between dorsal and pectoral fin; new scar (triangle shape) on the right side, close to mouth, and another one up the gills. New and clear scar with sucker marks; left side
WS13	Female	4.5	Adult	11/09/2019	Long scar in the left side of head, between eye and gills
WS11	Male	4	Adult	11/10/2019	Same long scar on the right side (50 cm; 2017), between dorsal and pectoral fin; new scar (triangle shape) on the right side, close to mouth, and another one up the gills. New and clear scar with sucker marks; left side
WS14	Male	3.5	Subadult	11/10/2019	Small scar on the right side of the body between the dorsal and pectoral fin
